# A Nonlinear Impact-Driven Triboelectric Vibration Energy Harvester for Frequency Up-Conversion

**DOI:** 10.3390/mi14051082

**Published:** 2023-05-20

**Authors:** Hadeel Abumarar, Alwathiqbellah Ibrahim

**Affiliations:** Department of Mechanical Engineering, University of Texas at Tyler, 3900 University Blvd., Tyler, TX 75799, USA

**Keywords:** frequency up, triboelectric, energy harvesting, up-conversion, transition, magnet

## Abstract

Energy harvesting effectively powers micro-sensors and wireless applications. However, higher frequency oscillations do not overlap with ambient vibrations, and low power can be harvested. This paper utilizes vibro-impact triboelectric energy harvesting for frequency up-conversion. Two magnetically coupled cantilever beams with low and high natural frequencies are used. The two beams have identical tip magnets at the same polarity. A triboelectric energy harvester is integrated with the high-frequency beam to generate an electrical signal via contact-separation impact motion between the triboelectric layers. An electrical signal is generated at the low-frequency beam range achieving frequency up-converter. The two degrees of freedom (2DOF) lumped-parameter model system is used to investigate the system’s dynamic behavior and the corresponding voltage signal. The static analysis of the system revealed a threshold distance of 15 mm that divides the system into monostable and bistable regimes. In the monostable and bistable regimes, softening and hardening behaviors were observed at low frequencies. Additionally, the threshold voltage generated was increased by 1117% in comparison with the monostable regime. The simulation findings were experimentally validated. The study demonstrates the potential of using triboelectric energy harvesting in frequency up-converting applications.

## 1. Introduction

Modern science and technology have advanced quickly, leading to a substantial rise in ultra-low-powered electronics across various industries. This increases public knowledge of ambient energies and creates a sizable market for them as potential sources to replace chemical batteries in these devices [1]. Using renewable energy sources, such as solar energy [2], radio frequency (RF) [3], thermal energy [4], and mechanical vibration energy [5], the principle of energy harvesting provides a way to power small devices. Mechanical vibrations occur naturally at low frequencies, as do ambient energies in our surroundings such as wind [6,7], ocean waves [8], human and vehicle movement [9,10], and working equipment, which has frequencies between 1 and 200 Hz [11].

There are a variety of mechanisms that have been utilized to harvest vibrational energy for electricity generation, including electrostatic transduction mechanisms [12,13], piezoelectric [14], electromagnetic [15,16], and triboelectric generators [17,18,19]. Triboelectric generators have some advantages for vibration energy harvesting since Wang’s team created the first triboelectric nanogenerator in 2012 [20]. These advantages include high output voltage, low cost, the flexibility of fabrication, and effective energy scavenging from low vibration frequencies [21,22]. Furthermore, triboelectric energy harvesters have shown great potential for capturing energy from a range of sources, such as human movement [23,24], mechanical vibration [25], smart home applications [26], wearable technology [27,28,29,30], implantable medical devices [31,32,33,34], and wireless sensor networks (WSN) [35,36,37]. Furthermore, triboelectric energy harvesting effectively converts small amounts of kinetic energy into electricity, typically in the microwatt range, under the influence of impact. Overall, triboelectric generators operate in four different ways, namely the in-plane sliding mode [38,39], the single electrode mode [40,41], the free-standing triboelectric layer mode [42,43], and the vertical contact separation mode [44,45] that have been used in this study.

Even though the harvested energy from the ambient vibrations appears to be high with high-efficiency conversion devices, some devices’ resonant frequencies do not match the frequency of the ambient vibrations or the bandwidth is limited to a specific range. As a result, if the device’s frequency deviates slightly from the harvester’s resonant frequency, the harvested power will drop significantly [46]. Additionally, according to its general mathematical formula, the energy harvester’s highest power is proportionate to the cube of its vibration frequency and decreases significantly at low frequencies [47]. Moreover, most environmental vibration energies in real-world situations are dispersed over narrow frequency ranges [48,49,50]. Therefore, researchers have spent considerable time and effort finding solutions to the above-mentioned issues [51]. Therefore, a frequency up-converter to transform low excitations into high-frequency oscillations was suitable for energy harvesting applications. However, frequency-up conversions were traditionally achieved either using contact mechanisms or non-contact impulse accelerations [52,53]. This led to the development of mechanical impact frequency up-converters with direct contact [54,55]. Frequency-up converters were used for energy harvesting applications utilizing different approaches such as snap-through buckling [56] and bistable oscillations [57]. Recently, Yin et al. [58] presented a shoe that harvests energy from human motion using a mounted piezoelectric energy harvester with frequency-up conversion. Furthermore, Atmeh et al. [14,59] investigated theoretically and experimentally a piezoelectric frequency up-converter mechanism for harvesting energy at ambient range utilizing high-frequency oscillations.

This paper proposed a frequency up-converter to harvest low-frequency vibrations utilizing a triboelectric generator and magnetic coupling. The frequency-up converter mainly consists of two cantilever beams coupled with two identical magnets attached to the tip of the beams. The tip magnets are attached facing each other at the same polarity to provide a nonlinear repulsive magnetic force responsible for converting low-frequency vibrations into high-frequency oscillations. A two-degree of freedom (2DOF) lumped parameter model has been used to investigate the beam’s dynamic behavior and the produced voltage signal. The rest of this article describes the device’s setup and demonstrates how the frequency-up-converter energy harvester operates. In addition, each beam’s static response and the frequency variations caused by the magnet separation distance and the generation of voltage and power at different values of the magnet separation distance are investigated. Experiments are also carried out to validate the simulation results derived from the theoretical model. In the end, the optimum operation region is determined.

## 2. Device Configuration and Principle of Operation

**Figure 1:**
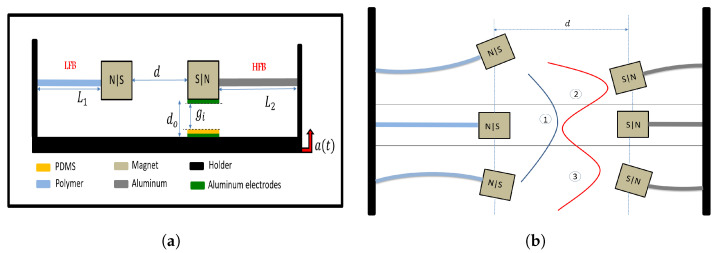
(**a**) 2D schematic of the energy harvester system; (**b**) Device operation under repulsive magnetic force.

The 2DOF triboelectric energy harvester consists of two cantilever beams, as shown in Figure 1a. The left side beam is made of polymer with a low natural frequency and is called, for simplicity, a low-frequency beam (LFB), while the right side beam is made of aluminum with a higher natural frequency and is called a high-frequency beam (HFB). For each beam, a magnet is attached to the tip, where the magnets face each other at the same polarity to provide a nonlinear mechanical repulsive magnetic force. The magnetic force transfers the energy from the high-frequency to low-frequency range achieving the concept of frequency up-conversion. $L_{1}$ and $L_{2}$ are the lengths of the (LFB) and (HFB), respectively. Both tip magnets attached to the beams are separated by the distance *d*. The whole setup is attached to a holder and installed in an electrodynamic shaker to provide an excitation source to the system. The triboelectric generator mainly consists of three layers: a lower fixed electrode made of aluminum with attached Polydimethylsiloxane (PDMS) insulator layer and an upper aluminum electrode attached to the bottom surface of the HFB’s tip magnet. The upper electrode and the insulator layer are separated by an initial gap $g_{i}$, while the two electrodes are separated by a distance $d_{0}$. Once the system starts vibrating under harmonic excitation a(t), the upper electrode periodically impacts the insulator, generating an electrical signal based on contact electrification and electrostatic induction [60].

Figure 1b depicts how the distance between magnets impacts the beam’s response and potential energy function. The the magnetic force varies based on the separation distance and generates an additional equilibrium point, resulting in a bistable system. The potential energy function can either be a single well or a double well, depending on the distance between magnets. For greater distances between magnets, the beam exhibits monostable behavior, oscillating about a single stable point, i.e., point 1. At shorter distances, the system has two potential wells, and the beam oscillates between two stable equilibrium points, namely points 2 and 3, indicating bistable behavior. The critical separation distance that divides the monostable and bi-stable systems is called the threshold distance, or $d_{th}$.

## 3. Theoretical Model

**Figure 2:**
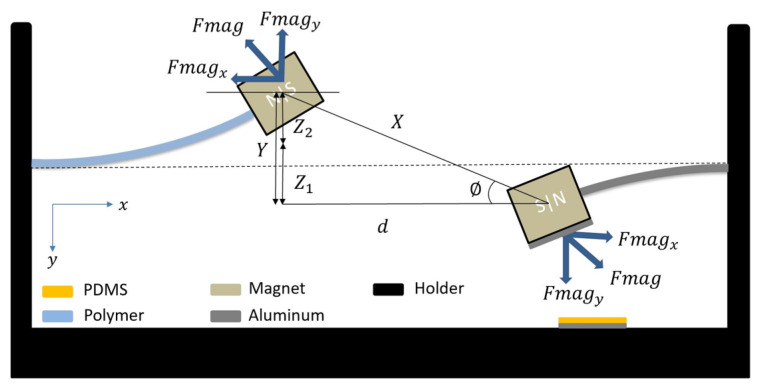
Magnetic interaction between the LFB and HFB.

Under mechanical excitation, the system will vibrate and generate electrical signal voltage due to the contact and separation between the harvester layers, Figure 2. Moreover, a repulsive force with the same magnitude will be induced between the two identical tip magnets, transferring the energy between both beams and given by Equation (1). Therefore, the total repulsive magnetic force can be analyzed into two components through the angle ϕ as shown in Figure 2. The first component is ($F_{magx}$) in the horizontal direction, which is assumed to have a minor effect on the transverse vibrations and balancing the longitudinal stiffness of the beam, and it will be neglected for simplicity. In contrast, the second component is ($F_{magy}$) in the vertical direction, which is assumed to be the only component responsible for the transverse deflections of the beams. Then, the transverse magnetic force will be as given in Equation (2). Where *X* is the distance between the centers of the two magnets ($X=\sqrt{d^{2}+Y^{2}}$), $F_{R}$ is the magnitude of the moments for the magnetic force and can be calculated from ($F_{R}=\frac{3\mu q_{1}q_{2}}{2\pi }$), where $q_{1}$ and $q_{2}$ are the moments of the magnetic dipoles for the tip magnets, μ is the permeability of the free space with a value of ($4\pi \times 10^{-7}\;mkg/s^{2}A^{2}$). Moreover, *d* is the horizontal separation distance between the tip magnets, and *Y* is the total deflection of the beams and is given by $Y=z_{1}\left(t\right)+z_{2}\left(t\right)$, where $z_{1}$ and $z_{2}$ are the deflection of the LFB and HFB, respectively.

(1) $$F_{mag}=\frac{F_{R}}{X^{4}}\sin\phi$$

(2) $$\begin{array}{c} F_{magy}=\frac{F_{R}Y}{{\left(d^{2}+Y^{2}\right)}^{5/2}} \end{array}$$

**Figure 3:**
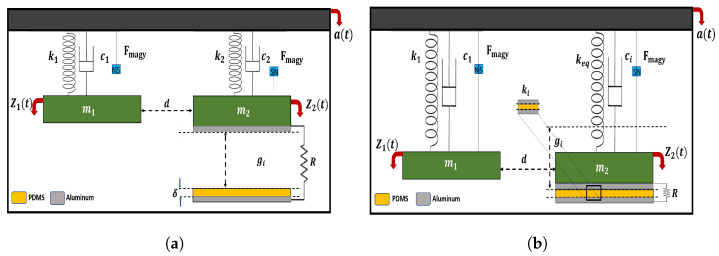
2DOF spring-mass-damper systems for the LFB and HFB: (**a**) before impact; (**b**) after impact.

A lumped parameter model of a two-degree of freedom (2DOF) system, shown in Figure 3, will be used to analyze the dynamic behavior and the generated electrical signal. Under vibration, the system will behave under two scenarios of motion, the first scenario is before impact, as shown in Figure 3a, and this occurs when the deflection of the HFB is not enough to reach the insulator layer (the tip magnet displacement less than the gap $g_{i}$). The second scenario is in the state of impact as shown in Figure 3b. The impact occurs when the upper electrode contacts the PDMS layer (the tip magnet displacement exceeds the gap $g_{i}$). This impact will increase the stiffness and the damping of the HFB, and this increment is expressed in $k_{i}$ and $c_{i}$ to be added to the system to represent the impact stiffness and impact damping, respectively. Accordingly, the governing equation of the system will be as follows [17]:

(3) $$\left\{\begin{array}{c} \begin{array}{cc} m_{1}{\ddot{z}}_{1}\left(t\right)+c_{1}{\dot{z}}_{1}\left(t\right)+k_{1}z_{1}\left(t\right)-F_{magy}=m_{1}a\left(t\right) & \\ m_{2}{\ddot{z}}_{2}\left(t\right)+c_{2}{\dot{z}}_{2}\left(t\right)+k_{2}z_{2}\left(t\right)+F_{magy}+F_{e}=m_{2}a\left(t\right) & z_{2}\left(t\right)<g_{i}\\ m_{2}{\ddot{z}}_{2}\left(t\right)+c_{i}{\dot{z}}_{2}\left(t\right)+k_{2}z_{2}\left(t\right)+k_{i}\left(z_{2}\left(t\right)-g_{i}\right)+F_{magy}=m_{2}a\left(t\right) & z_{2}\left(t\right)\geq g_{i}\\ \dot{q}=-\frac{q(t)}{\epsilon_{0}RS}\left(\frac{T}{\epsilon_{r}}+d_{0}-z_{2}\left(t\right)\right)+\frac{\sigma }{\epsilon_{0}RS}\left(d_{0}-z_{2}\left(t\right)\right) \end{array} \end{array}\right.$$

where a(t) is the harmonic base excitation (a(t) = A cos(Ωt)) with A is the amplitude, and Ω is the excitation frequency. The LFB’s equivalent mass and damping coefficient are given by $m_{1}$ and $c_{1}$, respectively, while $m_{2}$ and $c_{2}$ are the equivalent mass and the damping coefficient, respectively, of the HFB. $k_{1}$ is the equivalent stiffness of the LFB with tip magnet mass and is calculated by ($k_{1}=\frac{3E_{1}I_{1}}{L_{1}^{3}}$) [59], while $k_{2}$ is the equivalent stiffness of the HFB with tip magnet mass and is calculated by ($k_{2}=\frac{3E_{2}I_{2}}{L_{2}^{3}}$) [59]. In addition, the electrostatic force between the electrodes of the triboelectric energy harvester as they act as a parallel plate capacitor and is given by ($F_{e}=\frac{q^{2}\left(t\right)}{2\epsilon_{0}\epsilon_{r}S}$). Moreover, $k_{i}$ and $c_{i}$ are the stiffness and capacitance during the impact scenario of the HFB. The electrical charges (q(t)) are transferred between the two electrodes. Moreover, $\epsilon_{0}$ and $\epsilon_{r}$ are the permeability of the free space and the insulator, respectively. Finally, *S*, σ, and *T* are the surface area of contact, the surface charge density, and the thickness of the insulator, respectively. Accordingly, the following formula can be used to calculate both beam’s linear natural frequencies:

(4) $$\begin{array}{c} f_{i}=\frac{1}{2\pi }\sqrt{\frac{k_{i}}{m_{i}}}\;,\;i=1,2. \end{array}$$

where $k_{i}$ and $m_{i}$ are the effective mass and stiffness of the beams, respectively. The effective mass of the cantilever beam ($m_{heff}=0.375m_{b}$) [59], where $m_{b}$ is the beam mass.

It is worth mentioning that under the effect of the tip mass and base excitations enough to achieve the impact scenario as shown in the operation of the harvester, Figure 3, the upper electrode will come into contact with the lower PDMS electrode, generating electricity based on contact electrification and electrostatic induction. Under this condition, the beam will be bent, and the upper electrode will contact the lower insulator at a nonzero contact angle (not flat-to-flat surface contact). Therefore, a negative effect on the amount of energy harvested is expected. However, this issue is only shown at low excitations that result in a slight contact, while at high excitations, the inertia effect will be maximized, the two layers will contact each other perfectly, and the angle of contact will be almost zero. Moreover, we selected the material of the high-frequency beam (HFB) to be stiffer than the low-frequency beam (LFB) to achieve both high and low frequencies. However, we designed the HFB to be long to give extra flexibility in the structure and achieve the maximum possible contact between the triboelectric layers by minimizing the contact angle. Therefore, for simplicity, this effect has been neglected.

## 4. Experimental Setup

**Figure 4:**
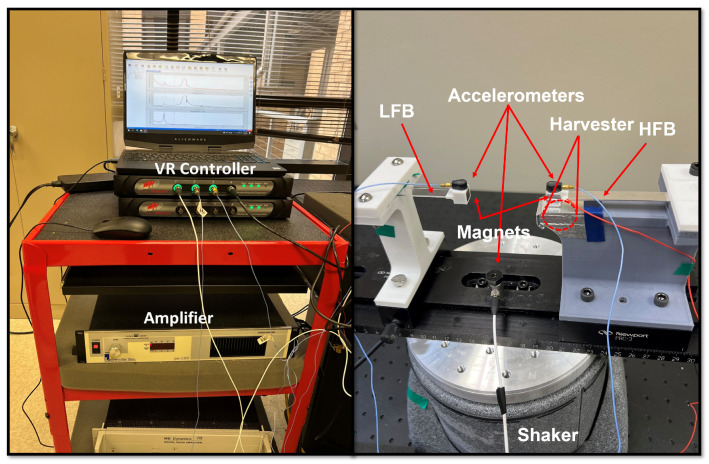
Experimental setup used to test the triboelectric energy harvester frequency-up converter.

To experimentally validate the numerical results from the theoretical model, we use the setup shown in Figure 4 to test the system under different excitation levels and frequencies. The main components of the setup are the VR9500 control unit, amplifier, electrodynamics shaker, and triboelectric energy harvesting system. First, the controller unit regulates the base excitation applied by the shaker to control its amplitude and frequency. Then, the control unit sends signals to the amplifier for amplification before transferring them to the shaker that transfers the base excitation to the triboelectric energy harvester. As the triboelectric energy harvester system is subjected to base excitations from the shaker, it oscillates, and an impact between triboelectric layers starts generating electricity. Moreover, the accelerometers mounted on the tip magnets of each beam are connected to the VR9500 control unit and measure the deflection of the LFB and HFB beams to extract the frequency response and voltage curves. Finally, using the parameter listed in Table 1, the static and dynamic behaviors of the system can be extracted numerically.

## 5. Results and Discussion

### 5.1. Static Analysis

Since the repulsive magnetic force affects both beams statically, it is essential to investigate the static response of both beams as a function of the magnet spacing distance. Therefore, the static analysis of both beams can be formulated by setting all-time derivatives in Equation (3) to zero and replacing the base excitation with the gravitational acceleration (*g*) to include the weight effect. The weight affects only the static response, while it does not affect the dynamic response other than introducing a new equilibrium position [61]. Therefore, the static equations are given as follows:

(5) $$\begin{array}{c} k_{1}z_{1s}-F_{magys}-m_{1}g=0 \end{array}$$

(6) $$\begin{array}{c} k_{2}z_{2s}+F_{magys}-m_{2}g=0 \end{array}$$

where $z_{1s}$ and $z_{2s}$ are the static deflections of the LFB and HFB, respectively. $F_{magys}$ is the static magnetic force in the transverse direction and is given by:

(7) $$\begin{array}{c} F_{magys}=\frac{F_{R}\left(z_{1s}+z_{2s}\right)}{{\left({\left(z_{1s}+z_{2s}\right)}^{2}+d^{2}\right)}^{5/2}} \end{array}$$

**Figure 5:**
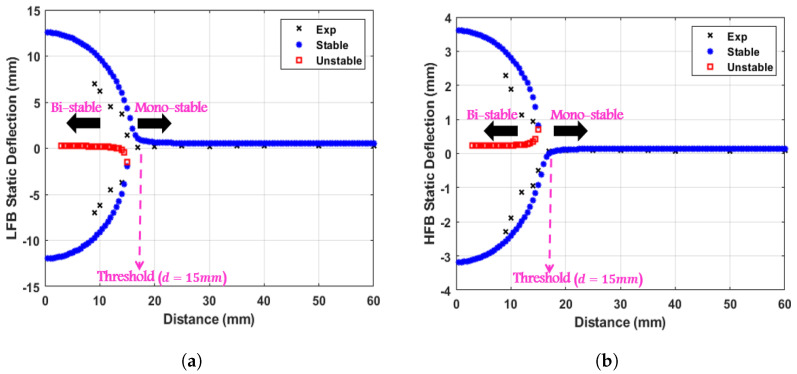
Experimental and theoretical static response of the (**a**) LFB, and (**b**) HFB. The threshold distance, $d_{th}$, was found to be 15 mm.

At large distances between the two magnets, the magnetic force is negligible. Therefore, each beam only undergoes a static equilibrium deflection due to the tip weight effect, which is also still considered very small. However, by lowering the separation distance between the two magnets to the threshold distance, both beams start to deflect from their previous equilibrium points to settle to new equilibrium points due to the effect of the magnetic force induced by lowering magnet spacing. By varying the distance between the two magnets, the corresponding static equilibrium points can be extracted numerically by solving for the roots of Equation (7) and experimentally by measuring the vertical deflections of both beams from the initial horizontal axis with a ruler. The experimental and theoretical variation of the static deflection for the LFB and HFB tip magnet centers with varying the distance between them are shown in Figure 5 with a good agreement. The maximum deflection values of the LFB and HFB are 12.6 and 0.38 mm, respectively. Both static responses showed a threshold separation distance $d_{th}$ of 15 mm that divides the static profiles into the monostable region ($d>d_{th}$) and bistable region ($d<d_{th}$). For the monostable regime, a single stable equilibrium solution is shown. In contrast, in the bistable regime, each static response has two stable (upper and lower) branches and one unstable branch (middle). The effect of the weight of each tip mass is shown as a symmetry-breaking bifurcation phenomenon at the threshold distance.

### 5.2. Dynamic Analysis

In this section, we will discuss how the magnetic force affects the natural frequency fluctuation of the nonlinear harvester. Toward this, the total deflections of the beams will be based on the static and dynamics deflections as follows:

(8) $$\begin{array}{c} \begin{array}{cc} \; & z_{1}=z_{1s}+z_{1u}\\ \; & z_{2}=z_{2s}+z_{2u} \end{array} \end{array}$$

where $z_{1u}$ and $z_{2u}$ are the dynamic deflection of the LFB and HFB, respectively. The total vertical deflection is:

(9) $$\begin{array}{c} \begin{array}{cc} \; & Y=Y_{s}+Y_{u}\\ \; & Y_{u}=z_{1u}+z_{2u} \end{array} \end{array}$$

Then, the magnetic force is:

(10) $$\begin{array}{c} F_{magy}=\frac{F_{R}\left(Y_{s}+Y_{u}\right)}{{\left(d^{2}+{\left(Y_{s}+Y_{u}\right)}^{2}\right)}^{5/2}} \end{array}$$

Using Taylor’s series around zero dynamic deflection $\left(Y_{u}=0\right)$, the magnetic force was expanded up to nine terms to avoid the complications of the magnetic formula in the numerical solution and guarantees the conversion of the numerical solution. This results in the following magnetic force:

(11) $$\begin{array}{c} \begin{array}{cc} \; & F_{magy}=F_{magys}+F_{magyu}\\ \; & F_{magy}=\frac{F_{R}Y_{s}}{{\left(d^{2}+Y_{s}^{2}\right)}^{5/2}}+\sum_{i=1}^{9}\alpha_{i}Y_{u}^{i}\left(t\right),\;i=1,2,\ldots ,9 \end{array} \end{array}$$

where $\alpha_{i}$ are the coefficients of Taylor’s series expansion of the dynamic magnetic force [59]. Then, by taking the first linear term of the expanded Fmagy and substituting it in Equation (3), the governing equation of the system will be:

(12) $$\left\{\begin{array}{ccc} & m_{1}{\ddot{z}}_{1u}\left(t\right)+c_{1}{\dot{z}}_{1u}\left(t\right)+\left(k_{1}-\alpha_{1}\right)z_{1u}\left(t\right)-\alpha_{1}z_{2u}\left(t\right)-F_{magyu}=m_{1}a\left(t\right) & \\ & m_{2}{\ddot{z}}_{2u}\left(t\right)+c_{2}{\dot{z}}_{2u}\left(t\right)+\left(k_{2}+\alpha_{1}\right)z_{2u}\left(t\right)+\alpha_{1}z_{1u}\left(t\right)+F_{magyu}+F_{e}=m_{2}a\left(t\right) & z_{2}\left(t\right)<g_{i}\\ & m_{2}{\ddot{z}}_{2u}\left(t\right)+c_{i}{\dot{z}}_{2u}\left(t\right)+\left(k_{2}+\alpha_{1}\right)z_{2u}\left(t\right)+\alpha_{1}z_{1u}\left(t\right)+k_{i}\left(z_{2u}\left(t\right)-g_{i}\right)+F_{magyu}=m_{2}a\left(t\right) & z_{2}\left(t\right)\geq g_{i}\\ & \dot{q}=-\frac{q(t)}{\epsilon_{0}RS}\left(\frac{T}{\epsilon_{r}}+d_{0}-z_{2u}\left(t\right)\right)+\frac{\sigma }{\epsilon_{0}RS}\left(d_{0}-z_{2u}\left(t\right)\right) \end{array}\right.$$

Accordingly, the nonlinear natural frequencies of the LFB and HFB can be calculated from the following formulas:

(13) $$\begin{array}{c} f_{1}=\frac{1}{2\pi }\sqrt{\frac{k_{1}-\alpha_{1}}{m_{1}}}\;f_{2}=\frac{1}{2\pi }\sqrt{\frac{k_{2}+\alpha_{1}}{m_{2}}} \end{array}$$

where $\alpha_{1}$ is the first linear term of the magnetic force after the expansion using Taylor’s series and is given by:

(14) $$\begin{array}{c} \alpha_{1}=\frac{F_{R}\left(d^{2}-4Y_{s}^{2}\right)}{{\left(d^{2}+Y_{s}^{2}\right)}^{7/2}} \end{array}$$

#### 5.2.1. Natural Frequencies

**Figure 6:**
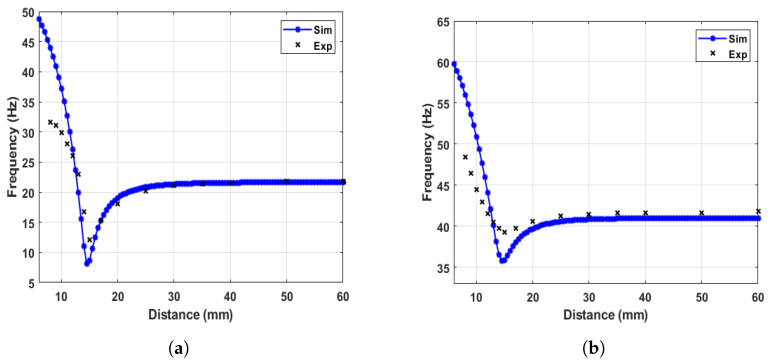
The simulated and experimental variations of the nonlinear natural frequency with magnet spacing for (**a**) LFB, (**b**) HFB.

**Figure 7:**
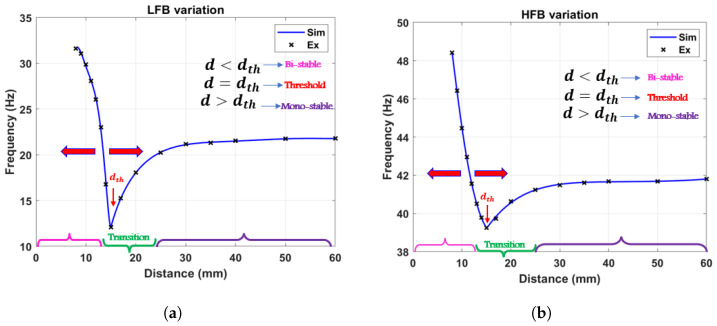
The simulated and experimental variations of the nonlinear natural frequency with magnet spacing using curve fit equations for (**a**) LFB, (**b**) HFB.

The variation of the nonlinear natural frequency with varying the magnets separation distance for both beams was extracted numerically using Equation (13) and experimentally by exciting the system at 0.05 g excitation level as shown in Figure 6. After that, the piece-wise curve fit functions were utilized to calculate the nonlinear natural frequencies variation with the magnets separation distance *d* for the LFB and HFB and then plotted as shown in Figure 7. The results show a threshold separation distance of 15 mm, consistent with findings from the static results in Figure 5. Moreover, the natural frequencies of both beams start to decrease to reach the minimum values at the threshold distance and then start to increase again with distances below the threshold distance. Moreover, the data in Figure 6 shows consistency between the simulation and experimental results in the monostable range, while it shows some discrepancies in the transition and bistable regimes. This discrepancy could be from different sources, such as the strong magnetic nonlinearity in the system, the lack of accuracy of the magnetic force equation used in the theoretical model, and the accuracy limitations of the lumped parameter modeling compared to the continuous modeling. Even though these discrepancies between the simulated and experimental results are minor, they would result in significant mismatch issues later when the dynamic behavior of the harvester is investigated. Therefore, to overcome these issues, the experimental results of the variation of the natural frequencies were used to extract the experimental stiffness values for both beams with curve fitting techniques to extract stiffness equations in piece-wise form as shown in Equations (15) and (16) for the LFB and HFB, respectively. The curve-fitting piece-wise equations are functions of the spacing distance between the two magnets. These stiffness equations were used to extract the variations of the natural frequencies for both the LFB and HFB and compared to the experimental results as shown in Figure 7. It is clearly shown that the previous discrepancies are removed, and it shows excellent matches for both LFB and HFB. Moreover, to enhance the accuracy of the analytical model while investigating the system’s dynamic behavior, the stiffness terms $\left(k_{1}-\alpha_{1}\right)$, and $\left(k_{2}+\alpha_{1}\right)$ in Equation (12) will be replaced by ($k_{LFB}$, and $k_{HFB}$), respectively.

(15) $$k_{LFB}=\left\{\begin{array}{cc} -21448+10055.9d-1844.1d^{2}+167.2d^{3}-7.5d^{4}+0.1d^{5} & \;\mathrm{if}\;d<d_{th}\\ -336.1+43.2d-1.6d^{2}+0.02d^{3}+4.0\times 10^{-5}d^{4}-4.0\times 10^{-6}d^{5}+2.5\times 10^{-8}d^{6} & \;\mathrm{if}\;d\geq d_{th} \end{array}\right.$$

(16) $$k_{HFB}=\left\{\begin{array}{cc} -227.5+468.5d-93.0d^{2}+8.0d^{3}-0.33d^{4}+0.01d^{5} & \;\mathrm{if}\;d<d_{th}\\ 112.3+26.8d-1.1d^{2}+0.02d^{3}-2.0\times 10^{-4}d^{4}+9.4\times 10^{-7}d^{5} & \;\mathrm{if}\;d\geq d_{th} \end{array}\right.$$

#### 5.2.2. Linear Results

**Figure 8:**
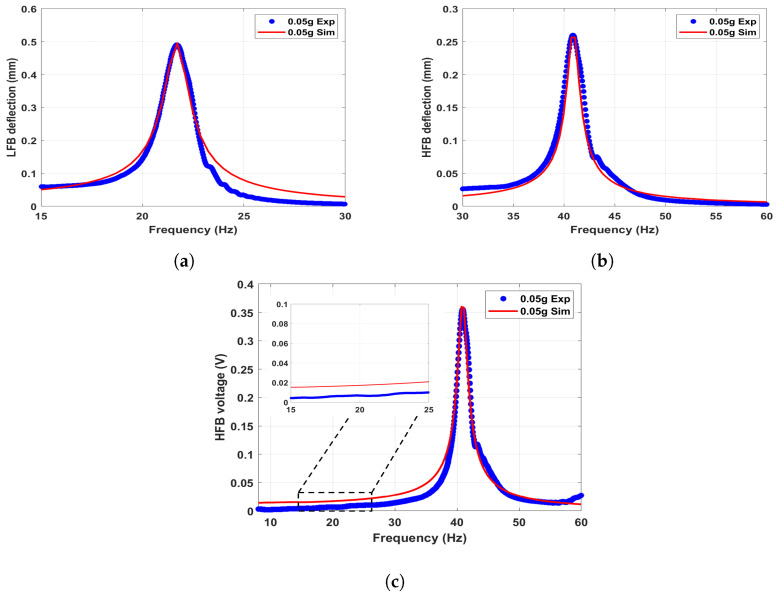
The linear simulated and experimental results at a low excitation level of 0.05 g: (**a**) frequency response curve of the LFB; (**b**) frequency response curve of the HFB; and (**c**) frequency voltage curve of the HFB. $c_{1}=0.04$, $c_{2}=0.039$, and σ=1μC/m${}^{2}$.

By removing the influence of the magnetic force ($F_{magy}=0$), the linear response of the system and the generated voltage can be extracted. The experimental and simulated frequency response curves of the LFB and HFB at a low excitation level of 0.05 g are shown with good agreement in Figure 8. It can be noticed that the linear natural frequencies of the LFB and HFB are found to be (21.8 Hz) and (41 Hz), respectively. Moreover, the corresponding frequency voltage curve for the HFB is shown in Figure 8c, reflecting a maximum output voltage of 0.35 V. Furthermore, by focusing on the low-frequency range in Figure 8c, it is clear that there is no generated electrical signal at the LFB range due to eliminating the magnetic effect, and no interaction between the beams occurs.

The periodic motion in the structure generates electric charges to the surfaces of triboelectric materials. The generated electrical charges depend on the surface charge density (σ), which is a function of the chemical properties of the materials and the micro-surface patterns that define the contact area [62]. Therefore, introducing micropatterns increases contact surfaces and enhances conversion efficiency [63]. Moreover, the amount of pressure applied on the triboelectric layers can play a role in the magnitude of the surface charge density. However, the surface charge density shows variations with time, so it is complicated to measure this value. Moreover, damping arises from removing energy by radiation or dissipation, and it is generally measured under cyclic or near-cyclic motion conditions. However, the damping factor varies as a function of frequency and excitations. Therefore, in our analysis and for simplifications, the surface charge density and damping values will be reported for each case separately.

#### 5.2.3. Nonlinear Results

The energy harvester’s dynamic behavior under the magnetic force’s effect at the monostable, transition, and bistable regimes will be examined in this section. The triboelectric generator was attached to the HFB, while the generated electrical voltage signal was monitored at the LFB range to achieve the concept of frequency up-converter due to the magnetic coupling effect. The generated frequency–voltage curves were extracted at the three regimes under different excitation levels to demonstrate the frequency up-converter concept and investigate the effect of the magnetic force on the system’s dynamic behavior.

**Figure 9:**
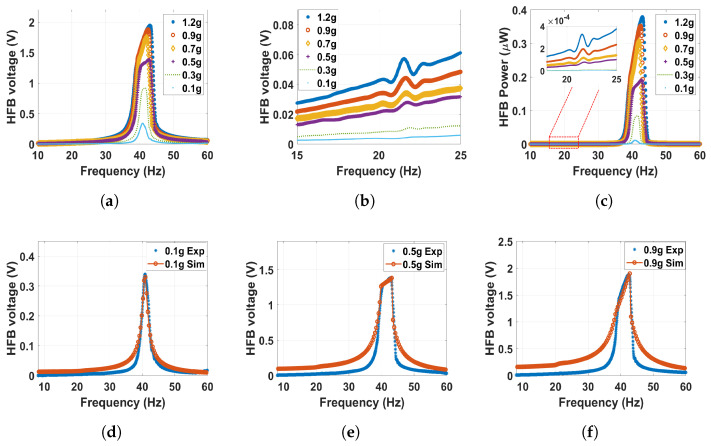
The frequency voltage curve of the HFB at different excitation levels with 60 mm separation distance: (**a**) experiment results in full range; (**b**) experiment results with zoom in at the LFB range; (**c**) experiment power results of the HFB; (**d**) experimental validation of the model at 0.1 g, $c_{1}=0.1$, $c_{2}=0.05$, and σ=3.5μC/m${}^{2}$; (**e**) experimental validation of the model at 0.5 g, $c_{1}=0.1$, $c_{2}=0.045$, and σ=5.6μC/m${}^{2}$; (**f**) experimental validation of the model at 0.9 g, $c_{1}=0.08$, $c_{2}=0.11$, and σ=5.15μC/m${}^{2}$.

The analysis will begin with the monostable regime and start with a large magnets separation distance of *d* = 60 mm. The corresponding experimental and theoretical results of the frequency–voltage and power curves under different excitation levels are shown in Figure 9. At the HFB range, the system acts linearly at a low excitation level. For example, it can be noticed that at a low excitation level of 0.1–0.3 g, the results are very close to the linear response results shown in Figure 8c. This is because the excitation level is very small, and the generated voltage is due to the capacitance effect between the triboelectric generator’s electrodes. However, increasing the excitation level above 0.3 g, an impact between the harvester layers starts at the HFB range (around 40 Hz), resulting in higher bandwidth and amplitudes. On the other hand, at the range of the LFB (around 22 Hz), even though the magnetic force is considered weak at this distance, the influence of the magnetic interaction is clearly shown by the new show-up voltage signal generated at this range as shown in Figure 9b. The newly generated voltage signal at the LFB range, even though the triboelectric generator was attached to the HFB, is proof of the concept of the frequency-up conversion. Furthermore, increasing the excitation level also leads to a noticed increment in the generated voltage and power signals, reaching a maximum value of 0.06 V at the 1.2 g excitation level. In contrast, a maximum value of 0.33 nW was achieved in the generated power at the same g level. However, the amplitude at *d* = 60 mm is minimal since the magnetic interaction is fragile at this large distance.

**Figure 10:**
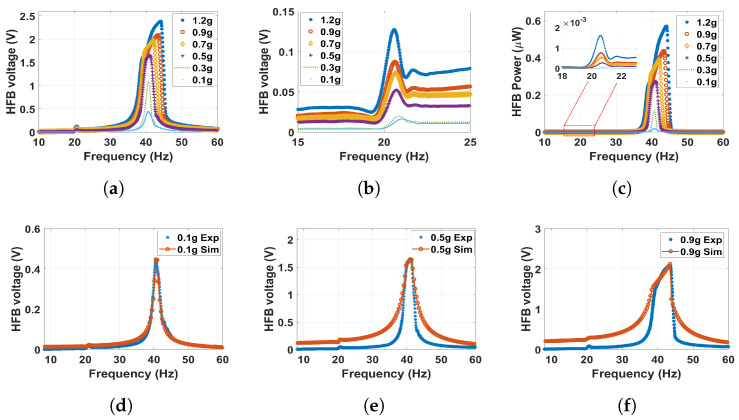
The frequency voltage curve of the HFB at different excitation levels with 30 mm separation distance: (**a**) experiment results in full range; (**b**) experiment results with zoom in at the LFB range; (**c**) experiment power results of the HFB; (**d**) experimental validation of the model at 0.1 g, $c_{1}=0.01$, $c_{2}=0.04$, and σ=3.9μC/m${}^{2}$; (**e**) experimental validation of the model at 0.5 g, $c_{1}=0.02$, $c_{2}=0.09$, and σ=6.9μC/m${}^{2}$; (**f**) experimental validation of the model at 0.9 g, $c_{1}=0.03$, $c_{2}=0.09$, and σ=6.7μC/m${}^{2}$.

**Figure 11:**
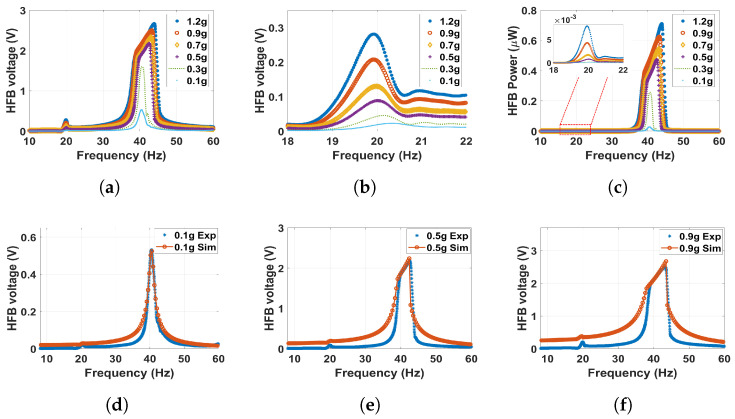
The frequency voltage curve of the HFB at different excitation levels with 25 mm separation distance: (**a**) experiment results in full range; (**b**) experiment results with zoom in at the LFB range; (**c**) experiment power results of the HFB; (**d**) experimental validation of the model at 0.1 g, $c_{1}=0.02$, $c_{2}=0.05$, and σ=5.5μC/m${}^{2}$; (**e**) experimental validation of the model at 0.5 g, $c_{1}=0.03$, $c_{2}=0.03$, and σ=7.5μC/m${}^{2}$; (**f**) experimental validation of the model at 0.9 g, $c_{1}=0.04$, $c_{2}=0.08$, and σ=8μC/m${}^{2}$.

In order to study the dynamic behavior in the monostable regime, but at a more substantial magnetic force effect, the distance between the two magnets was set to 30 mm. The outcomes are shown in Figure 10, where the experimentally extracted frequency voltage curves are well-matched with simulations of the theoretical model. Moreover, it can be noticed that the natural frequencies of both beams are slightly shifted to the left in comparison with the linear natural frequencies to reach approximately 21 and 40.6 Hz for the LFB and HFB, respectively, indicating a softening behavior. This softening behavior is due to the effect of changing the separation distance (d) in the term $\alpha_{1}$ according to Equations (13) and (14). Moreover, at higher excitation levels, the natural frequencies shifted more to the left to show significant softening behavior. This shift at higher excitation is due to the effect of the quadratic nonlinearity from the magnetic force. Moreover, the output voltage and power amplitudes increased with high excitation levels. The voltage at 1.2 g is maximized at 0.13 V at the LFB range, while the output power is maximized at 1.6 nW, which is higher than the values achieved at a 60 mm separation distance. After that, the separation distance is reduced to 25 mm. The corresponding matched experimental and theoretical frequency voltage and power curves are shown for different excitation levels in Figure 11. The results at this distance are close to the previous distance. However, we can notice here that the natural frequency is shifted more to the left, and the shifting increases with higher excitation levels to indicate a softening behavior. In addition, the magnetic force is magnified as the separation distance decreases, resulting in higher interaction between the two beams leading to a higher output voltage and power, which peaked at approximately 0.28 and 7.9 nW at the LFB range, respectively, which are significantly higher than the previous values.

**Figure 12:**
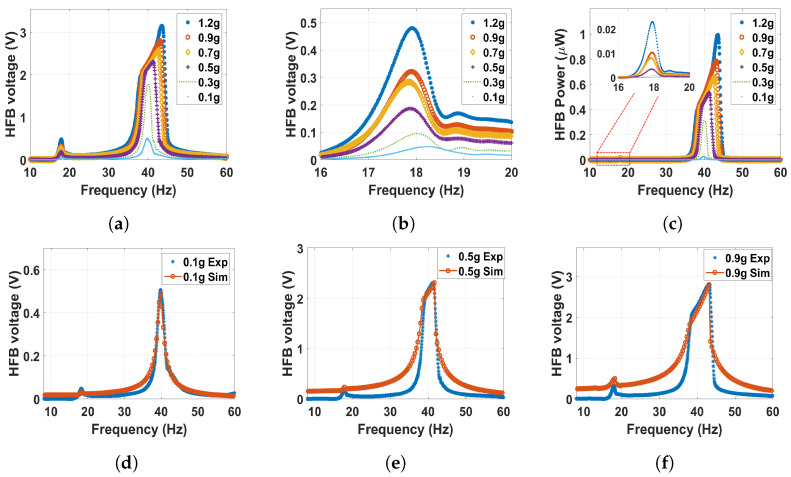
The frequency voltage curve of the HFB at different excitation levels with 20 mm separation distance: (**a**) experiment results in full range; (**b**) experiment results with zoom in at the LFB range; (**c**) experiment power results of the HFB; (**d**) experimental validation of the model at 0.1 g, $c_{1}=0.02$, $c_{2}=0.05$, and σ=5μC/m${}^{2}$; (**e**) experimental validation of the model at 0.5 g, $c_{1}=0.03$, $c_{2}=0.05$, and σ=8.5μC/m${}^{2}$; (**f**) experimental validation of the model at 0.9 g, $c_{1}=0.055$, $c_{2}=0.09$, and σ=7.9μC/m${}^{2}$.

Next, the separation distance decreased to 20 mm, and the frequency–voltage curves from both the experiment and simulation are in good agreement as shown in Figure 12. At this distance, the system will enter the transition regime from the monostable side. The results depicted in Figure 12 show that the natural frequency is shifted more to the left compared to the previous cases, reaching a lower value of 18.3 and 39.8 Hz for the LFB and HFB, respectively, which indicates a softening behavior. This shift results from the increased quadratic term of the magnetic nonlinearity at this distance in contrast to previous cases. In addition, it can be noticed in Figure 12b that the natural frequency of the LFB shifted more to the left with the increment in the excitation level, indicating a softening behavior, while increasing the excitation level to higher values will lead to a slight shift to the right indicating a hardening behavior. This exchange in the behavior is because at lower excitations, the quadratic nonlinearity is more dominant in the system, and a softening behavior is shown. On the other hand, when the excitation level increases, the cubic magnetic nonlinearity becomes more dominant, and a hardening behavior is shown. Moreover, as the excitation level increases, the output voltage rises due to the combined effect of the higher impact between the harvester layers at these higher excitation levels and the influence of the magnetic force. Furthermore, when compared to the monostable regime with a maximum output voltage of 0.48 V at the 1.2 g level of excitation, the output voltage at this distance increased by 700% in the LFB range. The last thing that can be noticed is that the power of the LFB at this distance peaked at 0.02 W at the 1.2 g excitation level as shown in Figure 12c, which is significantly higher than the values of the previous distances.

**Figure 13:**
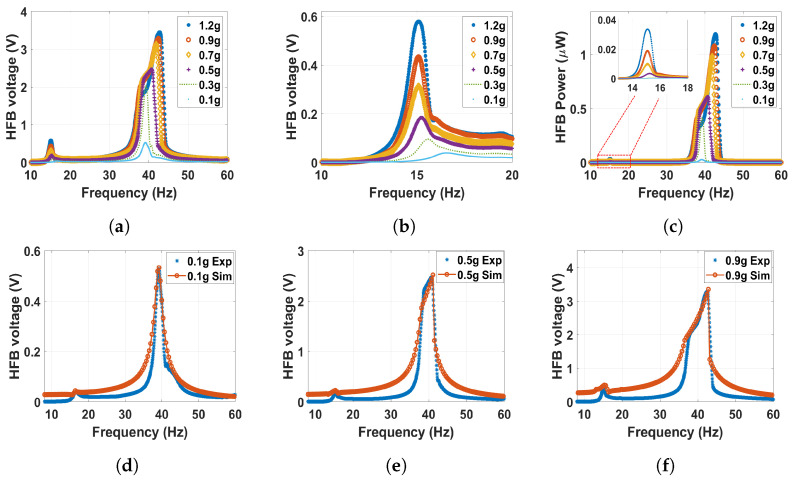
The frequency voltage curve of the HFB at different excitation levels with 17 mm separation distance: (**a**) experiment results in full range; (**b**) experiment results with zoom in at the LFB range; (**c**) experiment power results of the HFB; (**d**) experimental validation of the model at 0.1 g, $c_{1}=0.02$, $c_{2}=0.07$, and σ=7.5μC/m${}^{2}$; (**e**) experimental validation of the model at 0.5 g, $c_{1}=0.06$, $c_{2}=0.05$, and σ=8μC/m${}^{2}$; (**f**) experimental validation of the model at 0.9 g, $c_{1}=0.07$, $c_{2}=0.05$, and σ=8μC/m${}^{2}$.

Now, the separation distance is reduced to 17 mm, which is still in the transition regime but from the monostable side. The experiment and simulation’s frequency voltage and power curves are shown in Figure 13 with a good agreement. The natural frequencies are shifted to the left, indicating a nonlinear softening behavior because of the dominancy of quadratic nonlinearity. This significant shift is because the magnetic force between the two beams’ tip magnets becomes stronger in this case. Moreover, increasing the excitation level shifts the natural frequency to the right to achieve hardening behavior because of the dominancy of cubic nonlinearity. In addition, higher output voltage with an increment of 867% can be achieved in the LFB range compared with the monostable regime to reach a maximum value of 0.58 V at the 1.2 g excitation level. Finally, it can be noticed from Figure 13c that the generated power maximized at 0.033 W at the 1.2 g level of excitation.

**Figure 14:**
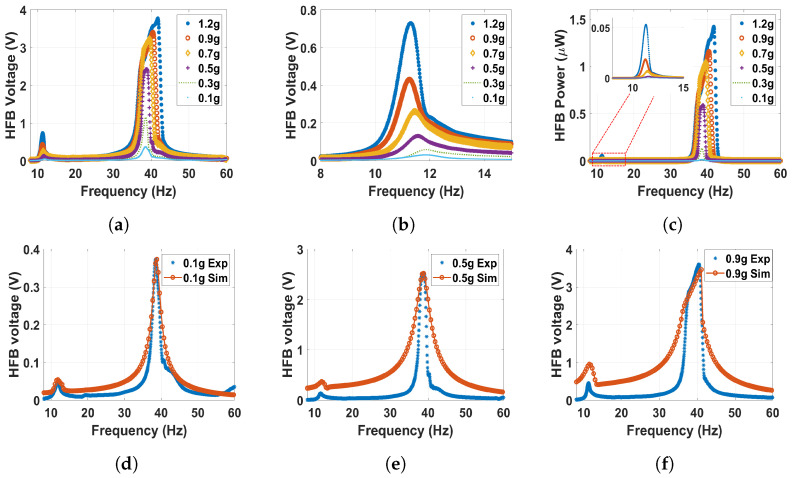
The frequency voltage curve of the HFB at different excitation levels with 15 mm separation distance: (**a**) experiment results in full range; (**b**) experiment results with zoom in at the LFB range; (**c**) experiment power results of the HFB; (**d**) experimental validation of the model at 0.1 g, $c_{1}=0.07$, $c_{2}=0.07$, and σ=5.2μC/m${}^{2}$; (**e**) experimental validation of the model at 0.5 g, $c_{1}=0.1$, $c_{2}=0.12$, and σ=12μC/m${}^{2}$; (**f**) experimental validation of the model at 0.9 g, $c_{1}=0.1$, $c_{2}=0.12$, and σ=10.7μC/m${}^{2}$.

**Figure 15:**
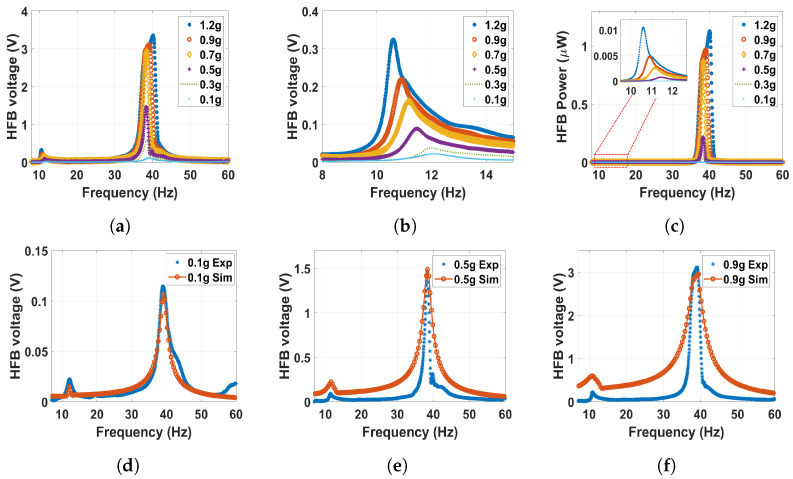
The frequency voltage curve of the HFB at different excitation levels with 14.5 mm separation distance: (**a**) experiment results in full range; (**b**) experiment results with zoom in at the LFB range; (**c**) experiment power results of the HFB; (**d**) experimental validation of the model at 0.1 g, $c_{1}=0.03$, $c_{2}=0.07$, and σ=1.5μC/m${}^{2}$; (**e**) experimental validation of the model at 0.5 g, $c_{1}=0.08$, $c_{2}=0.075$, and σ=4.4μC/m${}^{2}$; (**f**) experimental validation of the model at 0.9 g, $c_{1}=0.12$, $c_{2}=0.12$, and σ=8.2μC/m${}^{2}$.

By lowering the separation distance to reach the threshold at $d_{th}$ = 15 mm, the experimental and simulated frequency–voltage and power curves are shown in Figure 14. The maximum possible shift to the left in natural frequencies is achieved to reach the lowest value of 38.5 and 11.7 Hz for the HFB and LFB, respectively, which indicates a significant softening behavior. However, increasing the excitation level shifts the frequency to the right, indicating a hardening behavior. The maximum corresponding output voltage at the LFB range is 0.73 V at the 1.2 g level of excitation, where there is no increase in the output voltage signal compared with the previous distance at the same g level. Moreover, Figure 14c shows that the maximum power in the LFB range is 0.05 W at the 1.2 g level of excitation. The threshold distance achieved a maximum generated output voltage and power compared to all previous cases. Further decrease in the distance to 14.5 mm is still in the transition regime but from the bistable side. The frequency–voltage curves extracted experimentally agree with the ones extracted theoretically, as shown in Figure 15. The natural frequency for both beams shifted to the left in comparison with the linear natural frequency of each beam to reach 38.9 and 12 Hz for the HFB and LFB, respectively, indicating a softening behavior. Moreover, it can be noticed that the generated voltage dropped at this distance if it is compared with the threshold distance results in Figure 14. This is because the magnetic nonlinearity becomes very strong, producing less impact between the triboelectric layers, which means less output voltage. Therefore, higher excitation levels are needed to increase the impact between the triboelectric layers and generate electricity. However, the maximum voltage peaked at 0.33 V at the LFB range with the 1.2 g excitation level. However, the maximum power in the LFB range achieved at this distance is 0.01 W at the 1.2 g level of excitation as shown in Figure 15c.

**Figure 16:**
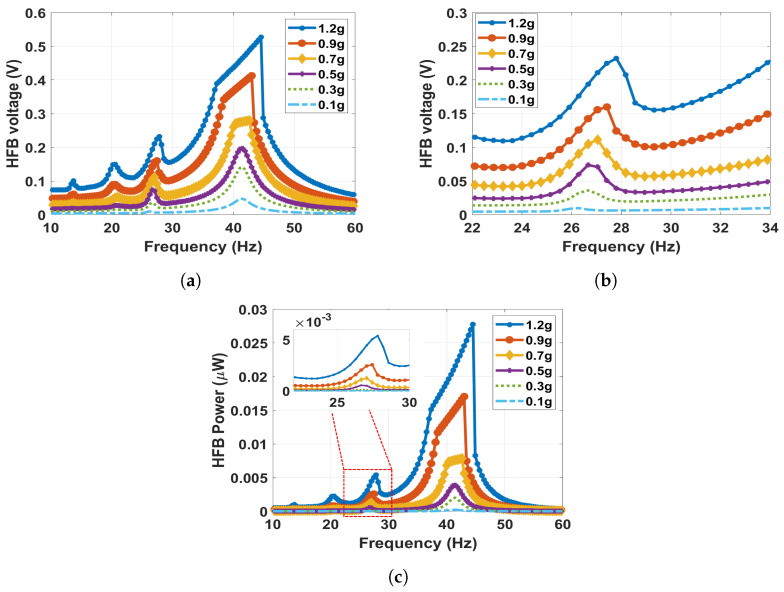
The frequency voltage curve of the HFB at different excitation levels with 12 mm separation distance: (**a**) simulated results in full range; (**b**) simulated results with zoom in at the LFB range, σ=1.7μC/m${}^{2}$; (**c**) simulated power results of the HFB.

Experimentally, at lower separation distances below 14.5 mm, the magnetic force becomes very strong and causes the beam to stick to the fixed electrode, which could lead to failure in the system and no electrical signal generated. Therefore, the dynamic behavior of the harvester for lower distances is extracted theoretically since we already validated our theoretical model in all the previous cases. In light of this, the distance decreased to reach 12 mm, and the results of the frequency voltage and power curves of the HFB with the zoomed response in the LFB range are shown in Figure 16. The results show that the natural frequencies are shifted to the right to higher values of 26.3 and 41.5 Hz for the LFB and HFB, respectively, indicating a hardening behavior. Moreover, it can be noticed that the output voltage and power dropped significantly to 0.23 V and 5.4 nW, respectively, in the LFB range in comparison with the previous case. This drop could be due to the system’s high nonlinearity, which makes the beam behave stiffer, and less impact between the triboelectric layers occurs, hence generating less voltage. Moreover, we can notice the appearance of the subharmonic resonance shown at ($\omega_{n1}/2=13.74$ Hz, $\omega_{n2}/2=20.58$ Hz) due to high nonlinearity and the dominance of the quadratic nonlinearity in the system.

**Figure 17:**
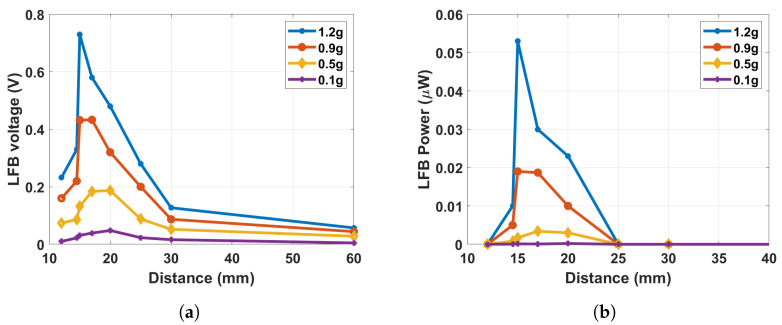
(**a**) The output voltage in the LFB range versus the separation distance at different excitation levels; (**b**) The output power in the LFB range versus the separation distance at different excitation levels.

To summarize the results discussed above and to specify the optimal operating regime, the output voltage and power at the LFB range were calculated and plotted versus the magnets’ separation distance at different excitation levels, as shown in Figure 17. The output voltage and power are maximized at the transition regime, particularly at the threshold distance at high excitation levels. Even though, at low excitation levels, the transition regime is still the most suitable for harvesting energy.

We utilized vibro-impact triboelectric transducers with magnetic nonlinearity to create a nonlinear frequency up-converter under harmonic excitations. This study focuses on combining magnetic nonlinearity with the inherent phenomenon of vibro-impact in triboelectric energy harvesters to transfer the energy between low and high oscillations. The combination of magnetic nonlinearity and vibro-impact is a novel strategy for triboelectric energy harvesters’ applications. Moreover, the addition of magnetic nonlinearity makes the harvester variable frequency energy harvester, where the operating frequency can be controlled by controlling the distance between the two magnets to target multiple applications at different frequency ranges.

## 6. Conclusions

In summary, a frequency up-converter using a vibro-impact triboelectric energy harvester efficiently converts low-frequency vibrations to a higher frequency is demonstrated. The system comprises low-frequency and high-frequency beams with two identical attached tip magnets. The magnetic coupling transfers the energy between the high-frequency and low-frequency ranges and generates a voltage signal at the LFB even though the generator was attached to the HFB, indicating a frequency up-converter. The structure was tested experimentally and validated with the simulated results of the theoretical model with good agreement. Different nonlinear behaviors of softening and hardening have been achieved by controlling the distance between the two magnets. Furthermore, a significant increment in the output voltage signal was achieved by lowering the distance between the two magnets. Moreover, the transition region was the optimum region to obtain the maximum voltage. Triboelectricity shows high efficiency in frequency up-conversion applications. 

## Figures and Tables

**Table 1:** Physical and geometrical parameters to be used in the modified model.

Parameters	Symbol	Value
LFB (length × width × thickness)	$L_{1}\times b_{1}\times h_{1}$	(38×10×1) mm
LFB Young’s modulus	$E_{1}$	2.344 Gpa
LFB Density	$\rho_{1}$	1220 kg/m${}^{3}$
LFB Damping coefficient	$c_{1}$	0.1 N.s/m
HFB (length × width × thickness)	$L_{2}\times b_{2}\times h_{2}$	(75 × 10 × 1) mm
HFB Young’s modulus	$E_{2}$	69.0 Gpa
HFB Density	$\rho_{2}$	2700 kg/m${}^{3}$
HFB Damping coefficient	$c_{2}$	0.1 N.s/m
Impact damping coefficient	$c_{i}$	3.4 $c_{2}$ N.s/m
Impact stiffness coefficient	$k_{i}$	3.4 $k_{2}$ N/m
Gap between Upper electrode and PDMS layer	$g_{i}$	0.001 m
Dimensions of PDMS layer (length × width × thickness)	$L_{P}\times b_{p}\times h_{p}$	(10 × 10 × 0.04) mm
PDMS dielectric constant	$\epsilon_{r}$	0.0001
Magnets side length	$L_{m}$	8 mm
Magnetic moment	$q_{1}=q_{2}$	0.5 A${}^{2}$/m
Resistance	*R*	10 MΩ
Permeability of free space	$\epsilon_{0}$	8.854 × 10${}^{-12}$

## Data Availability

Data available on request from the authors.
